# Insight into the anti-corrosion performance of capparis spinoza extract as a green corrosion inhibitor for carbon steel in hydrochloric acid environment

**DOI:** 10.1038/s41598-025-30968-5

**Published:** 2025-12-19

**Authors:** Osama A. Elgyar, Mohamed E. Yassen, Abdelfattah M. Ouf, Ahmed El-Hossiany, Abd El-Aziz S. Fouda

**Affiliations:** 1 Department of Chemistry, Faculty of Science, Mansoura University, Mansoura-35516, Egypt; 2 Higher Institute of Engineering and Technology, Tanta, Egypt; 3 Delta for Fertilizers and Chemical Industries, Talkha, 1179 Egypt

**Keywords:** Chemistry, Materials science

## Abstract

Capparis Spinoza extract (CPS) was evaluated as a green corrosion inhibitor for carbon steel (CS) in 1 M hydrochloric acid (HCl) solution. Weight loss (WL) measurement, potentiodynamic polarization (PDP), and electrochemical impedance spectroscopy (EIS) techniques were employed to assess its efficacy. CPS demonstrated excellent inhibition efficiency (% IE) By increasing both concentration and temperature. % IE reached 90.6% at 25 °C, 300 ppm, but it reached 95.2% at 45 °C and at the same concentration. Adsorption isotherm analysis indicated Temkin-type behavior, suggesting both physical and chemical adsorption mechanisms, with the latter being predominant. Thermodynamic calculations (-ΔG°_ads_ = -38.1 kJ mol^− 1^ at 25 °C) confirmed the spontaneous adsorption process, the stability of the formed adsorbed layer and shows that the CPS is mixed (physical and chemical) adsorbed on CS surface. PDP data classified CPS as a mixed-type inhibitor. EIS spectra exhibited characteristic inductive and capacitive loops at low and high frequencies, respectively. The formation of a protective CPS film on the CS surface was confirmed by atomic force microscopy (AFM) and Fourier transform infrared spectroscopy (FT-IR). The corrosion inhibition mechanism was elucidated through a combination of experimental and surface characterization techniques.

**Supplementary Information:**

The online version contains supplementary material available at 10.1038/s41598-025-30968-5.

## Introduction

 Corrosion is a slow, continuous, spontaneous process that occurs naturally”. Lately, “there have been significant financial losses, environmental harm”, and even potential concealed concerns to human safety as a result of metal corrosion^1^. “Thus, effectively preventing metal corrosion has long been the primary goal of concerned researchers. High carbon steel is a type of carbon steel that has excellent mechanical and flexible capabilities. It can be used to make wire products, plates, and body parts for automobiles. Sadly, mild steel rusted easily over extended periods of service due to exposure to harsh and complex environments^2^.

The authors selected hydrochloric acid (HCl) solution as the corrosive medium due to its widespread industrial relevance and aggressive corrosive nature. HCl is commonly used in acid cleaning, pickling of metals, descaling, and oil well acidizing operations, where metallic materials are frequently exposed to highly acidic environments^3^. Also, HCl, H_2_SO_4_ are particularly significant because they promote uniform and localized corrosion, allowing researchers to assess inhibitor efficiency, material resistance, and surface film stability under severe chemical stress.

The purpose of inhibitor is to stop CS corrosion. The inhibitors’ ability to establish an adsorbing layer on CS by physisorption or chemisorption is connected to their efficacy in inhibiting corrosion^4^. The kind of corrosive media, the chemical makeup of the inhibitors, and the kind and state of the metal surface all have an impact on the adsorption^5,6^. Good corrosion inhibitors are heterocyclic organic compounds with plenty of bonds and heteroatoms like O, N, and S that can be adsorbed on the metal surface^7^. The primary outcome of corrosion inhibition is the mitigation of potential hazards resulting from the thinnest metal in tanks and pipes which could cause material leak and catastrophic events like explosions and fires. Stated differently, the suppression of corrosion raises concerns regarding environmental protection and safety^8^. Plant extracts encompass a variety of organic compounds such as polyphenols, flavonoids, tannins, and alkaloids, which have established corrosion inhibition properties^9–13^. Numerous studies have explored the corrosion inhibition potential of plant extracts on metal and its alloys across different media. These extracts are typically derived from leaves, stems, seeds, or fruits of various plant species. Some commonly studied plant extracts for metal alloy corrosion inhibition include those from neem, aloe vera, green tea, mango leaves, and garlic^14–18^. By creating a barrier on the metal surface, plant extracts prevent corrosion by slowing down the corrosion process. The adsorption of phytochemicals onto the metal surface, which prevents corrosive substances from entering and stabilizes the passive oxide layer, may be one of the specific mechanisms behind inhibition^19–22^. Therefore, CPS extract is eco-friendly substance to inhibit the corrosion of CS in acidic media”.

*Capparis spinosa*, the caper bush, also called Flinders rose is a perennial plant that bears rounded, fleshy leaves and large white to pinkish-white flowers. *Capparis spinosa* (Caper) leaf extracts have been studied in Egypt for various medicinal properties, including antioxidant, anti-inflammatory, anti-diabetic, anti-cancer, and antimicrobial activities. The extracts are rich in bioactive compounds like flavonoids and phenolic acids, which are responsible for these beneficial effects (Figs. S1-S4 see supplementary file)). Studies have focused on the anti-carcinogenic properties of Caper leaf extracts against specific cell lines and their potential use for gastrointestinal issues. The below Table 1 shows list of *extract* sources used for corrosion inhibition of metals in different media.

**Table 1:** Lists of plant extracts used as corrosion inhibitors for metals and alloys in different solutions.

Plant	Medium	Metal	% IE	Ref.
*Capparis Spinoza leaves extract*	0.5 M HCl	high carbon steel	97.0	^23^
*Capparis spinosa *L. Extract	1.0 M HNO_3_	Copper	82.7	^24^
*Capparis Spinosa Extract*	1.0 M HCl	Carbon steel	93.19	^25^
*Capparis spinosa L. Extract*	1.0 M NaOH	copper	79.3	^26^
*aqueous extract of Ammi isnaga (L.) Lam (AE-AV)*	3 % NaCl	Cu-Zn alloy	91.67	^27^
*Essential oil from aerial parts of the Ruta Graveolens L. (RG-EO))*	1 M HCl	Mild Steel	95.3%	^28^
*Artemisia argyi*, *Chrysanthemum indicum*, and *Centipeda minima* extract	0.5 M H2SO4	Q235 steel	96.29%,96.50%, 97.52%, Respectively	^29^
*Carob fruit extract*	3.5 % NaCl	Cu-10 Ni, Cu-30 Ni	92.6%, 83.2%, respectively	^30^
*Capparis Spinoza extract (CPS)*	1 M HCl	carbon steel	90.6 %	Our results

The novelty statement for a green inhibitor (such as a CPS extract) is the claim that the study closes a knowledge gap by offering a new, environmentally friendly, and efficient solution and/or by offering previously unheard-of, comprehensive information on its protective mechanism, thus contributing both theoretically and practically to sustainable corrosion control.

The present study offers an advantage by providing a detailed investigation of corrosion behavior in HCl medium, emphasizing the effectiveness of the proposed protection method under realistic acidic conditions using different methods [weight loss (WL) method, (PDP), and (EIS)]. This not only contributes to understanding the underlying corrosion mechanisms but also supports the development of more durable and sustainable corrosion control strategies for industrial applications.


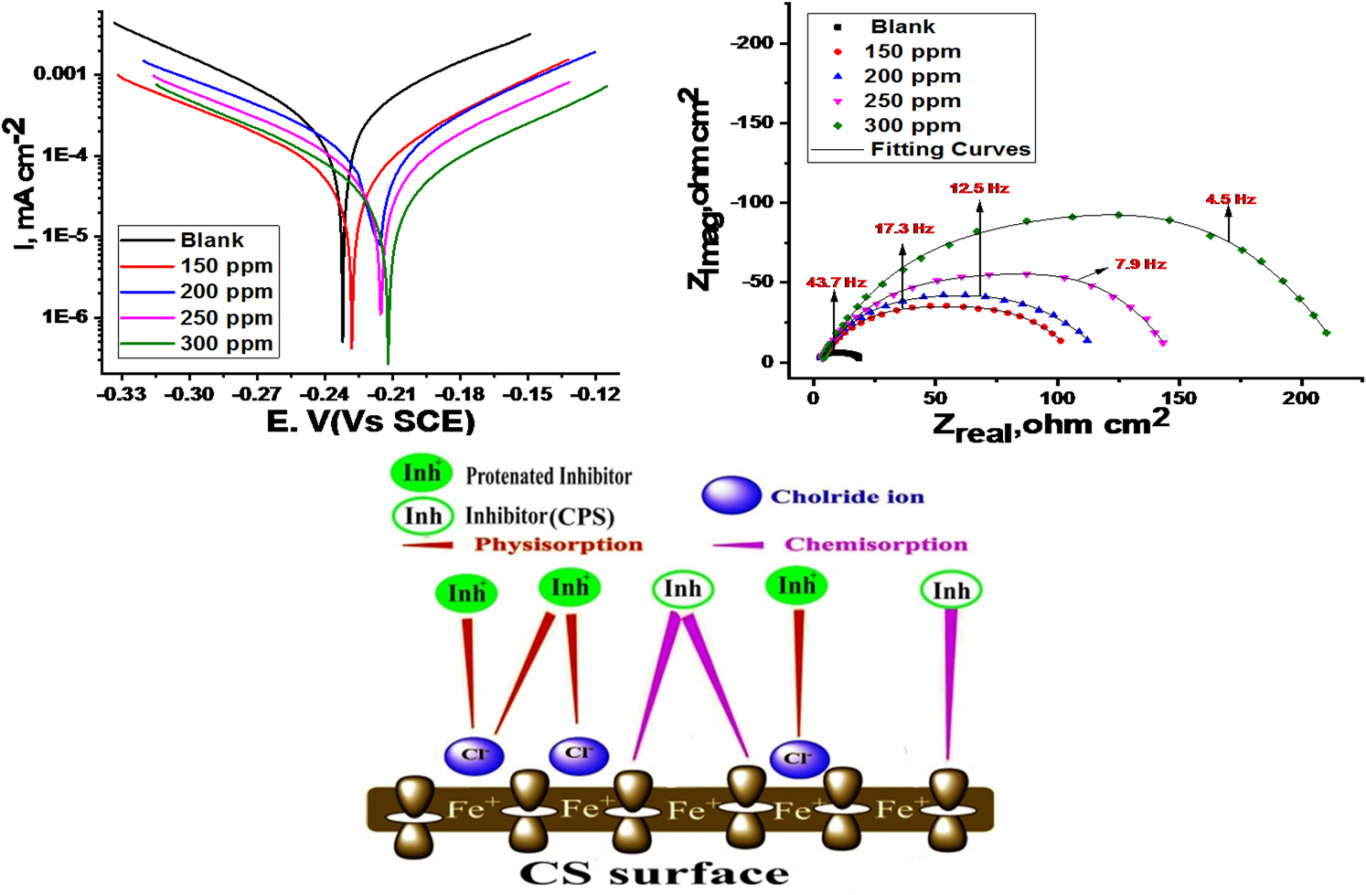


## Experimental techniques

### Materials and solutions

“The CS (1018) sheets which were used in this study had the following chemical composition, expressed as a weight%”: “0.0003 Silicon, 0.024 Phosphorus, 0.35 Manganese, 0.20 Carbon, and Fe the remaining percentage. By diluting the 37% hydrochloric acid utilizing bidistilled water, a 1 molar of the corrosive solution was created. A 1 cm by 1 cm work surface was the sole part of the CS electrodes that was not sealed with epoxy and submerged in the corrosive test solution (HCl 1.0 mol./L). The CS electrode was sequentially polished using the emery sheets (grades 250–1200) before to electrochemical tests”. After rinsing with bidistilled water, the polished CS was allowed to cool before being dried with cold air.

### Preparation of CPS extract

After being cleaned with deionized water, the (CPS) were dried. CPS was subsequently ground into tiny fragments. “Ten grams of dried leaves were weighed and placed to a 150 mL conical flask. The mixture was then mixed with one hundred milliliters of 70% ethanol. After being soaked for 48 hrs at 25 °C, the mixture was shaken for four hours at 25°C. After filtering the mixture through filter paper, it was kept at 0–5°C for later testing. Following the filtering process, the CPS extract was vacuum-concentrated to a precise volume of around 100 mL, and the extract dose was determined. A 1000 ppm stock solution was made by mixing one gram of the extract with one thousand milliliters of bi-distilled water. The extract used in the study was prepared by diluting CPS extract with bidistilled water, and its dose varied from 50 to 300 ppm”. The literature on the chemical components of CPS extract indicates that ferulic acid (c), p-coumaric acid (b), Syring aldehyde^31^. The toxicity of *CPS* extract is generally low under normal conditions. *Capparis spinosa* extract contains a diverse array of bioactive compounds, including flavonoids, alkaloids and other components like tocopherols, carotenoids, and various phenolic acids. These compounds are found in different parts of the plant, including the flowers, fruits, leaves, stems, and roots, and contribute to its various medicinal properties, such as antioxidant, anti-inflammatory, and antimicrobial effects. Quercetin, part of a subclass of flavonoids called flavonols, has received considerable attention because of its.

beneficial impact on health. Its biochemical activity is well documented. It is one of the most potent antioxidants among polyphenols^32^. Quercetin has also been to demonstrate to display the antiviral, antibacterial, anticarcinogenic and anti-inflammatory effects^33^. Also, is a strong antioxidant due to its ability to scavenge free radicals and bind transition metal ions. These properties of quercetin allow it to inhibit lipid peroxidation^34^. Its structural formula is presented in Scheme 1.

**Figure:**
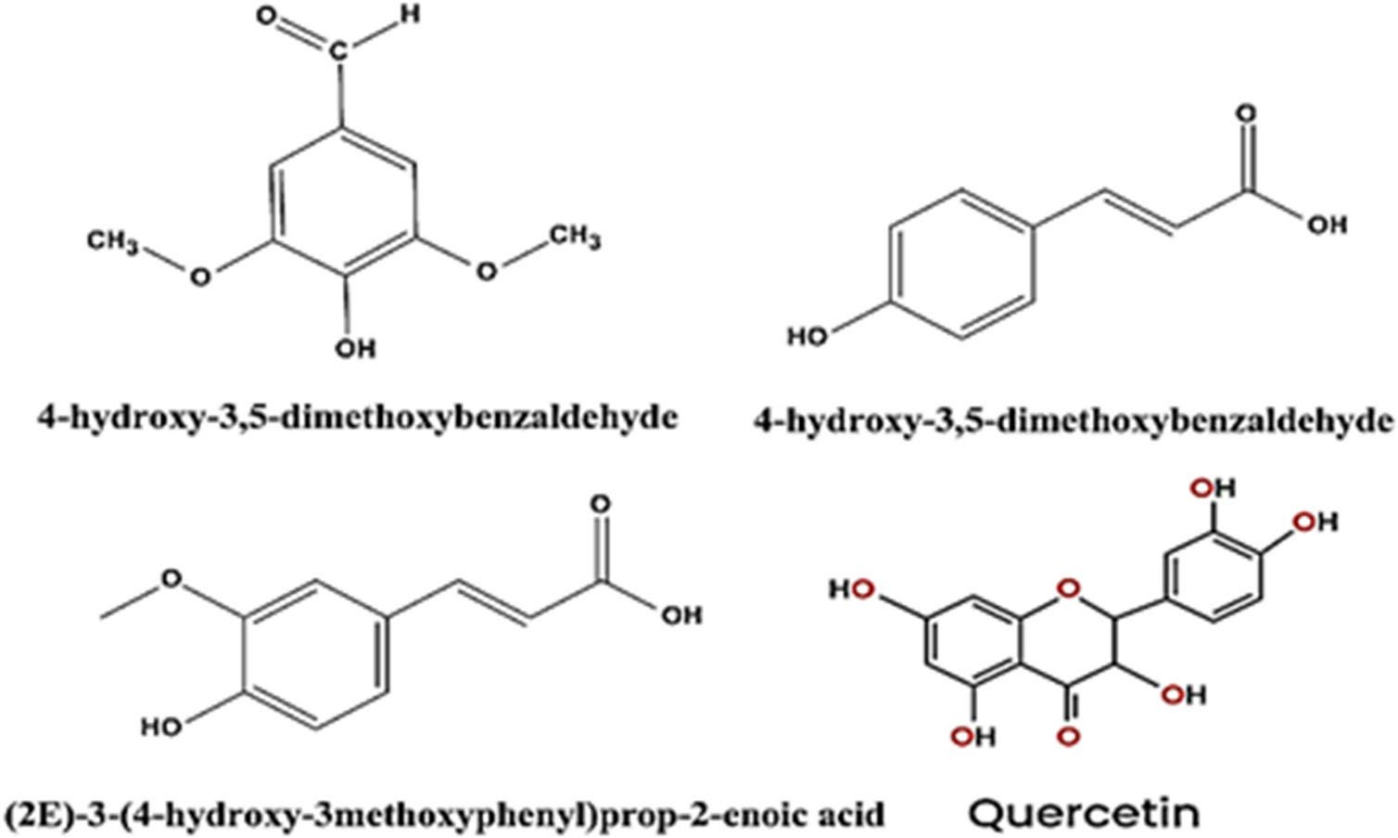
**Scheme 1: **List of some phytochemical constituents isolated from CPS extract

### WL experiment

The CS coupons used in this WL method were established in accordance with the ASTM G 31–72^35^ standard. “The CS samples sized at 2 cm x 2 cm x 0.01 cm, the CS specimens underwent mechanical polishing and abrading emery sheets with a grade range of 250–1200. After being cleaned utilizing bi-distilled water and acetone, they were dried using filter sheets in accordance with standard procedure. The specimens were weighted and then immersed in 100 mL solution in 150 mL beakers with varying concentrations of CPS extract and 1 M HCl at temperatures between 25 and 45°C. The CS specimens were removed from the solution, cleaned, dried, and weighed again after different durations” (30 to 180 min)^36^. All experiments were carried out in stagnant aerated solutions. The following equation was used to determine the metal sample’s corrosion rate (CR)^37^:

1 $$\:CR=\:\frac{\varDelta\:W}{AT}$$

where A (cm^2^) is the area of the specimen, t (min) is the time of immersion, and ∆W (mg) is the weight reduction. The surface coverage (Ɵ) and inhibition effectiveness (% IE) can be derived as described below^38^:

2 $$\% IE = \theta \times 100 = \left( {1 - \frac{{\Delta {W_1}}}{{\Delta {W_2}}}} \right) \times 100$$

where ∆W_1_ represents WL while the extract is present and ∆W_2_ represents WL when it is not.

### Electrochemical measurements

“The Gamry device cell (PCI4/750). Potentiostat./Galvanostat./ZRA comprises three electrodes”: “a SCE reference electrode, the counter electrode, which is a platinum wire, and a CS serving as the working electrode. A 1 × 1 centimeter square-shaped sample was connected electrically using a copper wire weld. It was then placed inside a Teflon tube and secured with an adhesive to function as a working electrode. Similar to the WL method, the electrode’s surface underwent treatment. Every electrochemical measurement was run at 25°C. The testing was started after allowing the CS to stabilize for 30 min prior to measurement. Tafel curves were developed using PDP analysis, applying potentials between − 1.5 and 0.5V versus OCP with sweep rate of 0.2 mVs^[─ 1^. Using the Tafel extrapolation method, the values of the corrosion potential (E_corr_) and corrosion current density (i_corr_) were calculated”. The PDP test yielded the following values for ʔ and % IE, as indicated below^39^:

3 $$\:\% {\text{IE = }}\theta \:\:{\text{x100 = }}\left[ {\:{\text{1 - }}\frac{{{{\text{i}}_{{\text{corr}}\left( {{\text{inh}}} \right)}}}}{{{{\text{i}}_{{\text{corr}}\left( {{\text{free}}} \right)}}}}} \right]{\text{x}}\:{\text{100}}$$

“where i_corr(inh)_ represents the density of the corrosion current while the extract is present and i_corr(free)_ represents the density of the corrosion current when it is absent. The equation Ɵ = [1 – (i_corr(inh)_/i_corr(free)_)] is derived from the postulate that the inhibitor operates via a blocking mechanism. The EIS tests were run at the OCP over the frequency range 10^5^ – 10^− 2^ Hz with a signal amplitude perturbation of 10 mV”. Below is the equation used to determine the values of θ and % IE from EIS tests^40^:

4 $$\:\% IE = \:\:x100 = \left[ {\:1 - \frac{{{R_{ct\left( {free} \right)}}}}{{{R_{ct\left( {ich} \right)}}}}} \right]x100$$

where the charge transfer resistance in the blank solution is denoted by R_ct(free)_, and the charge transfer resistance in the inhibited solution by R_ct(inh)_. To determine the impedance parameters R_ct_ and C_dl_, the impedance data was fitted to a suitable equivalent circuit. All tests were conducted in air-exposed stationary solutions. We conducted experiments in triplicate to ensure the reliability of our results.

## Surface morphology

### AFM examination

An effective method for examining the form of metal surfaces at the nano and micro scales is the AFM technique. This analysis’s ability to measure surface roughness is its main benefit. The 2 × 2 cm CS specimen was prepared as previously described in the WL method, and it was then submerged in 1.0 M HCl for three hours with and without a 300 ppm extract from CPS. The Japanese-made Shimadzu Wet-SPM (Scanning Probe Microscope) type was used for AFM testing.

### FT-IR spectroscopy analysis

FT-IR investigations were carried out to determine the spectrum inhibitors structure that produces peaks with values in order to “learn more about the functional groups” of the extract both before and after its adsorption on CS. Following the WL technique, the 2 × 2 cm CS specimen was prepared, immersed for 3 h in 1 M HCl containing 300 ppm CPS extract and then directly investigated utilizing FT-IR spectra by (ATR-IR Affinity-1, Shimadzu, Japan).

## Results and discussion

### WL measurements

**Fig. 1:**
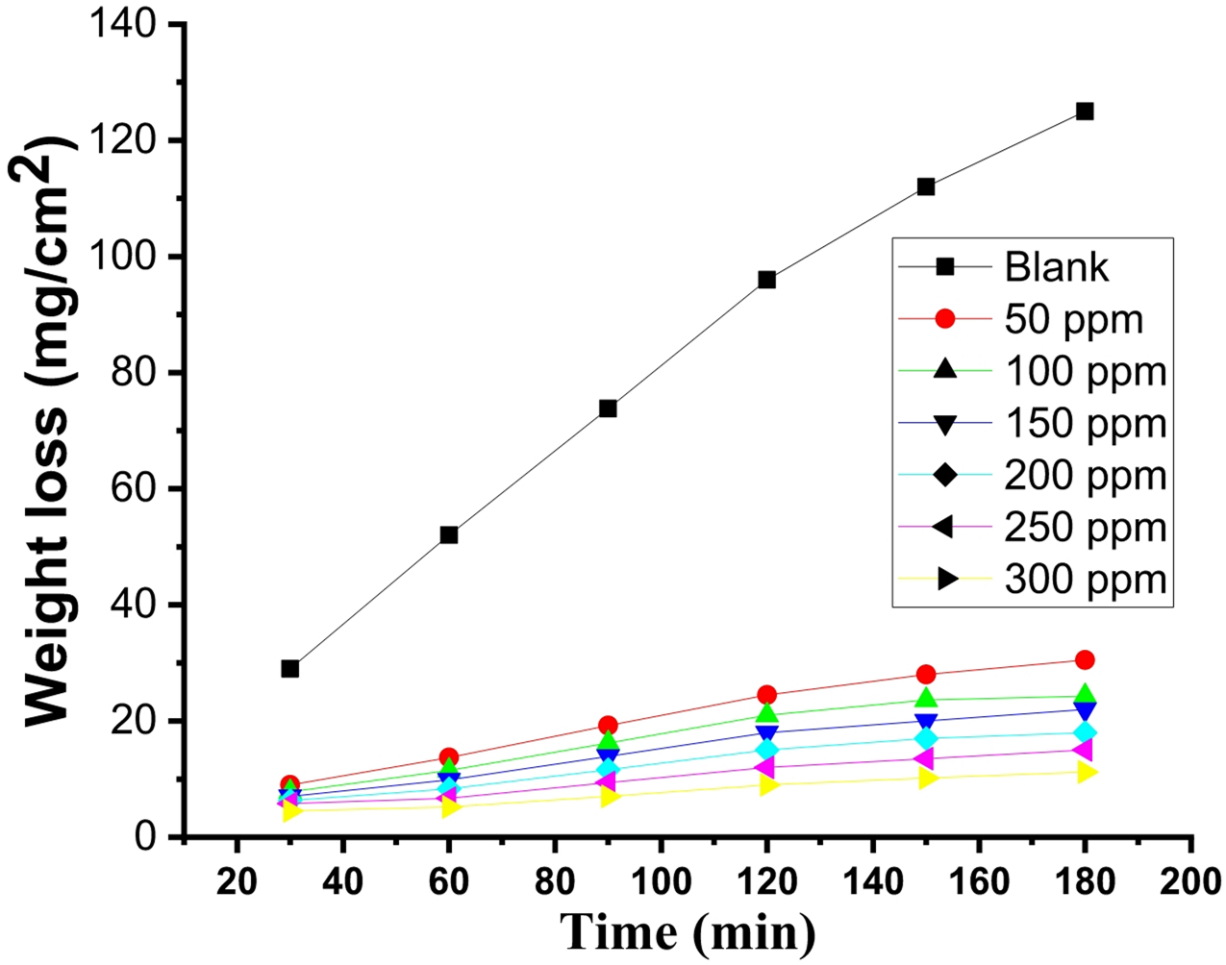
**“**Impact of increasing dose of CPS extract on WL at 25 °C for CS in 1 M HCl”.

“The effect of adding varying amounts (50–300 ppm) of CPS extract on CS corrosion in a 1.0 M HCl solution was evaluated utilizing the WL method”. “The association between the impact of various doses of CPS extract on CS surface WL- time curves at 25 °C was depicted in Fig. 1. It is evident that the WL of CS with the extract present was significantly lower than the WL in the free extract solution. As the extract dose grow, the layer created by the extract molecules adsorbed on the metal surface causes the WL to drop. This layer protects the metal’s corrosive sites and keeps the metal from corroding in the corrosive environment. The WL-time curves are roughly linear, signifying that the CS surface is free of oxide films^41^. Exceeding the optimal extract dose of 300 ppm results in solution saturation. Additionally, the possibility exists of powerful interactions between extract molecules anchored to the metal and those within the solution, as reported in the literature^42^ This interaction may induce desorption of the adsorbed extract layer into the solution”.

### Temperature effect

Using WL tests, the impact of different temperatures (25–45 °C) on “CS corrosion in 1.0 M HCl with and without varying extract doses was investigated”. “Table 2 displays the evaluations of CR and % IE derived from the WL tests for varying extract doses in 1 M HCl solution at altered temperatures. The findings showed that a rise in the dose of the extract leads to a reduction in CR value and a growth in % IE. This is an outcome of the extract’s growing degree of adsorption and subsequent covering of the CS surface as extract dose increases”. “The percentage of IE that increases as temperature rises shows that the extract may be chemisorbed on the CS surface”^43^.

**Table 2:** Variations of CPS extract dosages and their impact on k_corr_, θ and % IE of CS in 1 M HCl at different temperatures.

Temp., ^o^C	Conc., ppm	CR, mg cm^ـــ2^ min^ـــ1^	Ө	% IE
25	Blank	0.8000 ± 0.0015	----	----
	50	0.2041 ± 0.0011	0.745	74.5
	100	0.1750 ± 0.0012	0.781	78.1
	150	0.1500 ± 0.0010	0.813	81.3
	200	0.1250 ± 0.0012	0.844	84.4
	250	0.1000 ± 0.0015	0.875	87.5
	300	0.0750 ± 0.0016	0.906	90.6
30	Blank	0.9920 ± 0.0017	----	---
	50	0.2250 ± 0.0016	0.773	77.3
	100	0.1958 ± 0.0015	0.803	80.3
	150	0.1658 ± 0.0012	0.833	83.3
	200	0.1458 ± 0.0011	0.853	85.3
	250	0.1166 ± 0.0014	0.882	88.2
	300	0.0858 ± 0.0017	0.913	91.3
35	Blank	1.6670 ± 0.0018	----	---
	50	0.3458 ± 0.0013	0.793	79.3
	100	0.2958 ± 0.0019	0.823	82.3
	150	0.2583 ± 0.0015	0.845	84.5
	200	0.2083 ± 0.0020	0.875	87.5
	250	0.1583 ± 0.0012	0.905	90.5
	300	0.1250 ± 0.0014	0.925	92.5
40	Blank	2.3330 ± 0.0017	----	---
	50	0.4583 ± 0.0016	0.804	80.4
	100	0.3825 ± 0.0015	0.836	83.6
	150	0.3458 ± 0.0014	0.852	85.2
	200	0.2666 ± 0.0017	0.886	88.6
	250	0.2000 ± 0.0015	0.914	91.4
	300	0.1500 ± 0.0017	0.936	93.6
45	Blank	3.3170 ± 0.0019	----	---
	50	0.5750 ± 0.0018	0.827	82.7
	100	0.4833 ± 0.0017	0.854	85.4
	150	0.4166 ± 0.0014	0.874	87.4
	200	0.3166 ± 0.0013	0.905	90.5
	250	0.2166 ± 0.0012	0.935	93.5
	300	0.1608 ± 0.0011	0.952	95.2

“Using the Arrhenius relationship, we can estimate the energy of activation (E_a_^*^) as follows”^44^:

5 $$\log {\text{CR}} = \log A - \left( {\frac{{E_{\text{a}}^*}}{{2.303RT}}} \right)$$

**Fig. 2:**
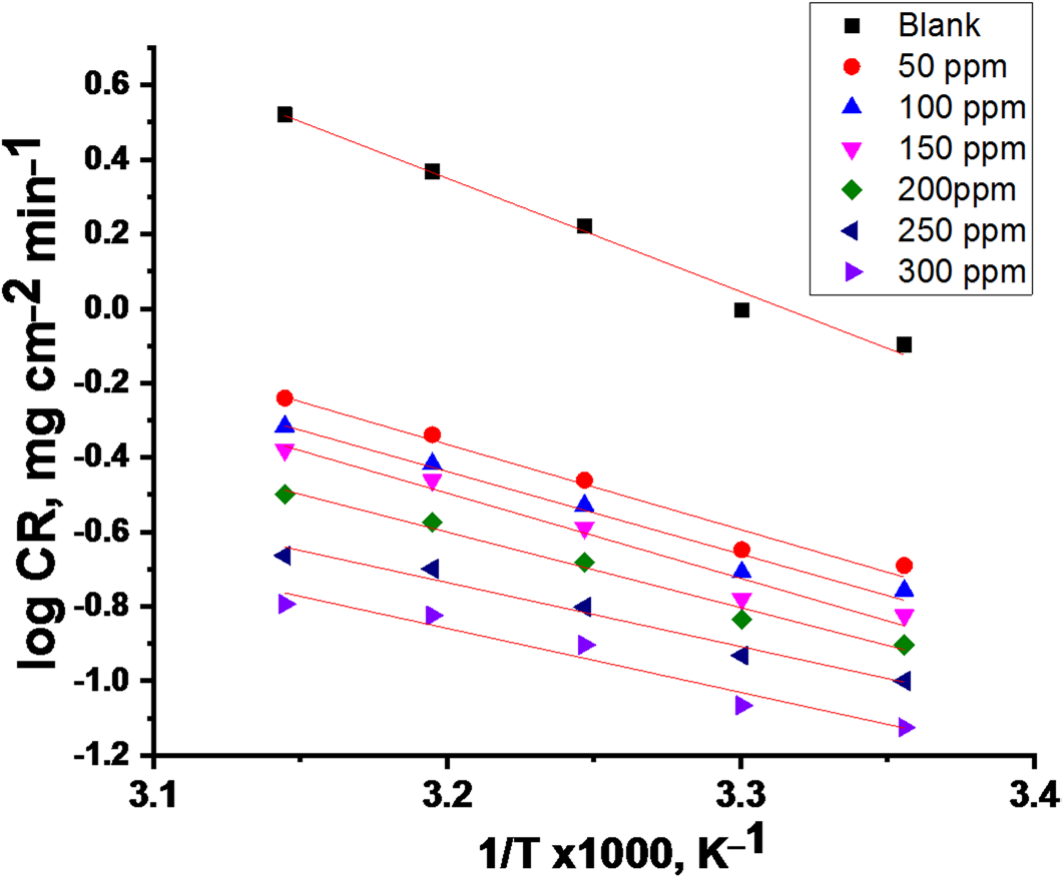
Log “CR” vs. “1000/T” for CS in a 1 M HCl solution with and without CPS extract.

A is the pre-exponential multiplier. As displayed in Fig. 2, “the log C.R against 1/T plots attendance and without doses of CPS extract produced straight lines with slopes from which the E_a_^*^ values were obtained”. “The next equation was used to calculate ΔH^*^ and ΔS^*^ of activation for the corrosion of CS^[“ 45^:

6 $${\text{CR}} = \left( {\frac{{RT}}{{Nh}}} \right)\exp \left( {\frac{{\Delta {S^*}}}{R}} \right)\exp \left( {\frac{{ - \Delta {H^*}}}{{RT}}} \right)$$

**Fig. 3:**
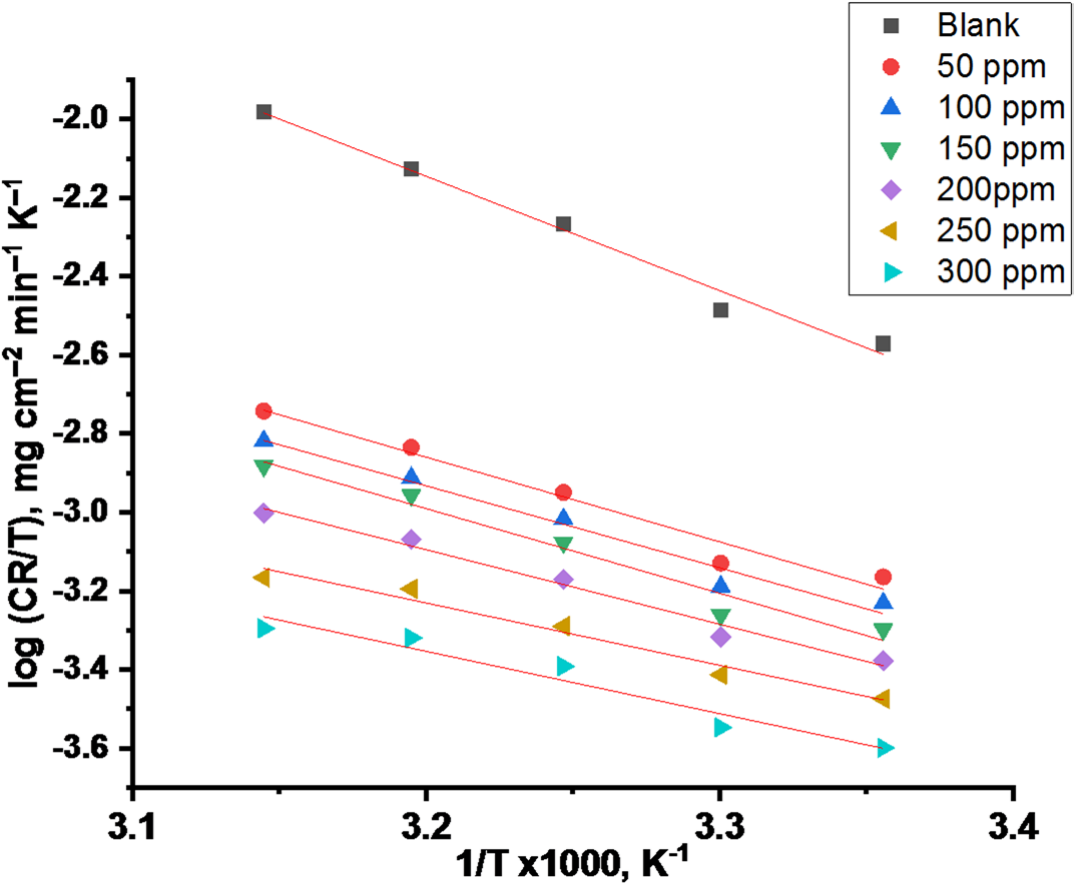
Log “CR/T” vs. “1000/T” for CS in a 1 M HCl solution with and without CPS extract.

“Plot (1/T) vs. log (CR/T) produced linear graphs (Fig. 3) and from their slopes and intercepts”, “the values of ΔH^*^ and ΔS^*^ were obtained, respectively. Table 3 contains the computed values for E_a_^*^, ΔH^*^, and ΔS^*^, both with and without the extract present. The results in Table 3 indicates that the values of E_a_^*^of the molecules adhered to the metal surface through chemical adsorption. It is suggested that disorder arises when the reactant changes into the activated complex due to the negative ΔS^*^ values, which showed that an association phase rather than a dissociation phase is included in the activated complex within the rate-limiting step^46^. The positive ΔH values indicate that the adsorption of CPS molecules onto the CS surface is endothermic. A decrease in ΔH^*^ in the presence of the extract compared to the blank solution suggests a lower energy barrier for the corrosion reaction”. The close agreement between E_a_^*^ and ΔH^*^ values (differing by approximately RT) supports a single-step, single-molecule dissolution mechanism for CS in the acidic medium^47^.

**Table 3:** **“**Values of E_a_^*^, ΔH^*^ and ΔS^*^ for CS in a 1 M HCl solution in presence and absence of CPS extract”.

Extract conc. ppm	E_a_^*^kJ mol^─1^	ΔH^*^kJ mol^─1^	ـــΔS^*^J mol^─1^K^─1^
Blank	58.2 ± 0.1609	55.6 ± 0.1622	60.3 ± 0.1452
50	43.8 ± 0.1809	41.2 ± 0.1241	120.3 ± 0.1241
100	42.5 ± 0.2009	39.8 ± 0.1112	125.9 ± 0.2144
150	43.6 ± 0.2117	41.1 ± 0.1012	122.8 ± 0.1104
200	42.1 ± 0.1421	36.1 ± 0.1457	140.6 ± 0.1327
250	33.7 ± 0.1432	30.2 ± 0.1678	162.2 ± 0.1471
300	32.9 ± 0.1247	30.2 ± 0.1754	164.5 ± 0.1120

### Isotherms of adsorption

The information required to comprehend the corrosion mechanism was obtained by the analysis of adsorption isotherms. “The Temkin adsorption isotherm equation (Eq. 7)^48^ is the most appropriate isotherm to fit the data after applying the different adsorption isotherms.” This isotherm demonstrates that the part of the CS surface that is protected by the inhibitor, θ, is determined by the inhibitor dose, C_inh_.

7 $$\ln K{C_{{\text{inh}}}} = a\theta$$

**Fig. 4:**
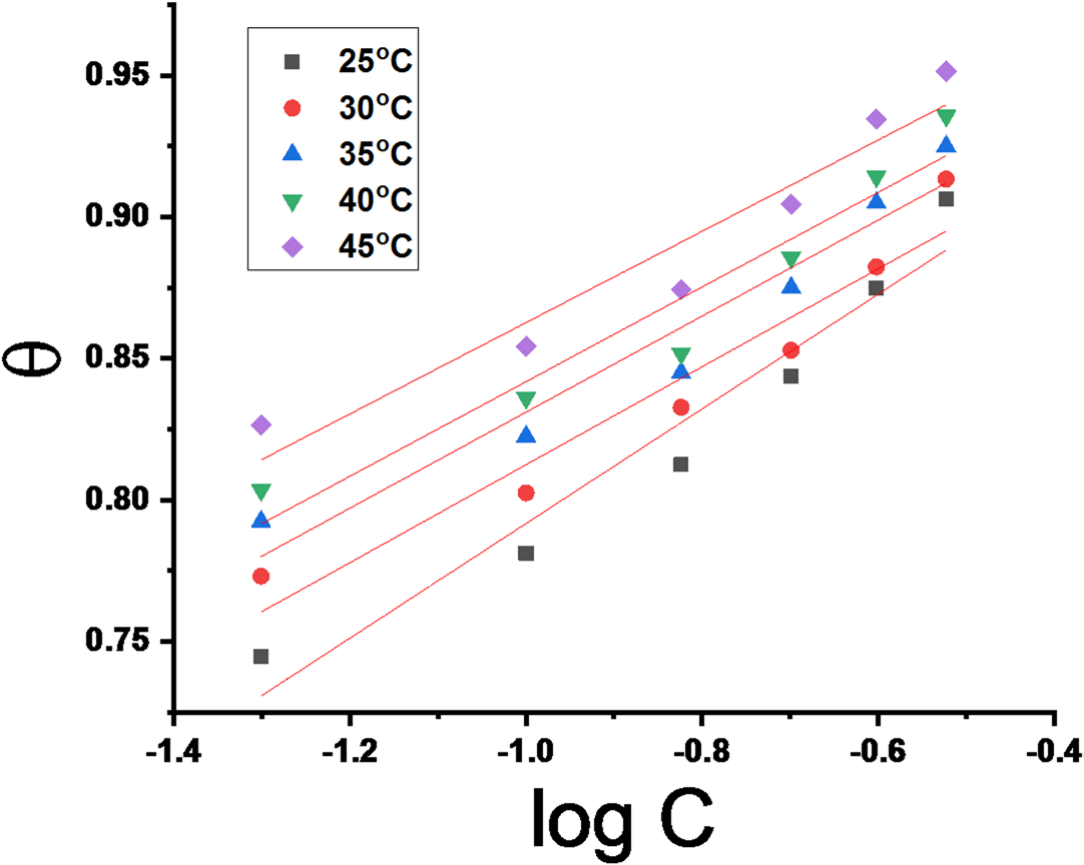
**“**Temkin isotherm for adsorption of CPS extract on the surface of CS in a 1 M HCl solution at different temperatures”.

K_ads_ represents the equilibrium constant for adsorption in the adsorption–desorption process. Three presumptions underpinned the Temkin adsorption isotherm: (i) a single layer of the adsorbate was adsorbed at a predetermined number of adsorption locations; (ii) there was no lateral interaction or steric hindrance among the adsorbed molecules; and (iii) all adsorption points on the adsorbent had constant activation energy, adsorption, and enthalpy. Temkin isotherm diagrams at different temperatures result in straight lines with R^2^ almost equal to unity, as seen in Fig. 4. The adsorption’s free energy (ΔG^o^_ads_)^49^ values were obtained using the following equation:

8 $${K_{{\text{ads}}}} = \left( {\frac{1}{{55.5}}} \right)\exp \left( {\frac{{ - \Delta G_{{\text{ads}}}^\circ }}{{RT}}} \right)$$

where 55.5 is the dose of water, in mol/L, at the metal/solution interface. Adsorption free energy (ΔG°_ads_) values were calculated, considering water concentration (55.5 mol/L) at the metal/solution interface”. The negative ΔG°ads values in Table 4 indicate spontaneous and stable adsorption of the extract onto the CS surface. Based on general guidelines, adsorption is considered physical for values around − 20 kJ/mol and chemical for values below − 40 kJ/mol. The calculated ΔG°_ads_ values between − 38.1 and − 49.3 kJ/mol suggest a predominantly chemisorptive mechanism^50^. Enthalpy of adsorption (ΔH°_ads_) was determined using^51^:

9 $$\log {K_{{\text{ads}}}} = \frac{{ - \Delta H_{{\text{ads}}}^\circ }}{{2.303RT}} + {\text{constant}}$$

**Fig. 5:**
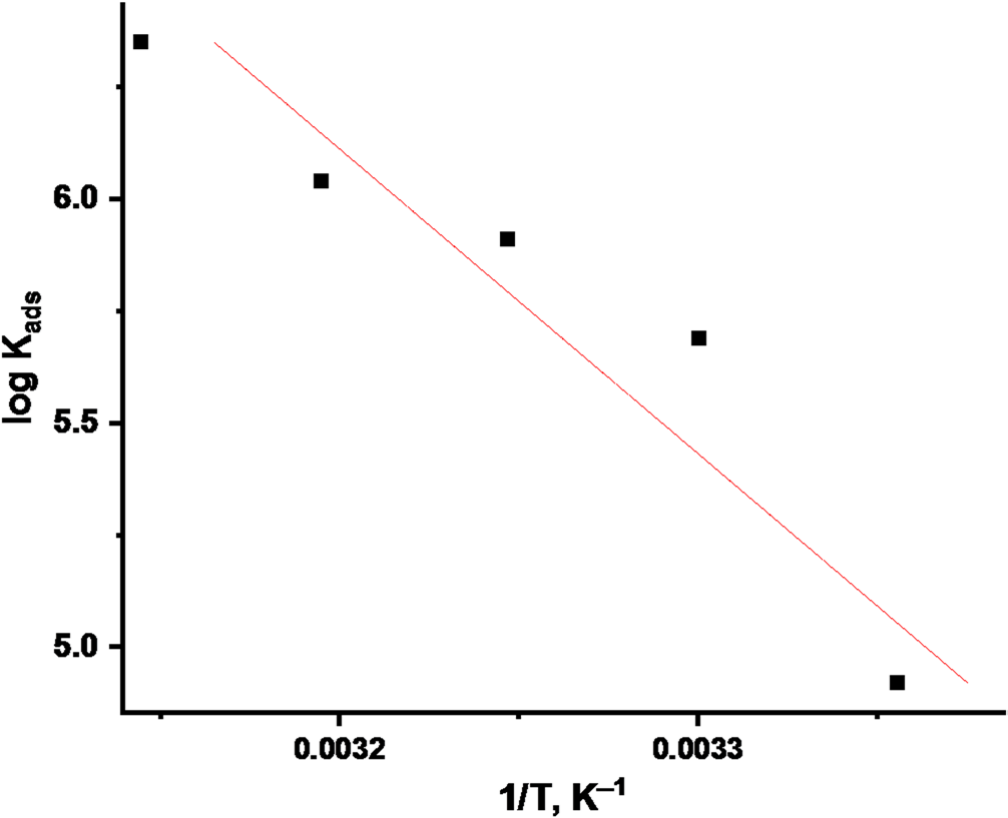
log K_ads_ vs. 1/T plots for CS dissolving in 1 M HCl.

“Plots log K_ads_ vs. 1/T for dissolving of CS in a 1 M HCl solution contains CPS is displayed in” Fig. 5. show straight line with regression constant R^2^ = 0.9139 “offerings positive ΔH^o^_ads_ data, suggesting an endothermic adsorption process”. “This elucidates the reason for the experimental results where the IE rises with temperature”. Finally, the entropy of adsorption (ΔS^o^_ads_) can be found using the following formula^25^:

10 $$\Delta S_{{\text{ads}}}^\circ = \frac{{\Delta H_{{\text{ads}}}^\circ - \Delta G_{{\text{ads}}}^\circ }}{T}$$

The negative signs of ΔS° indicate reduced disorder upon transition from the reactant to the adsorbed metal. This supports the potency and adsorption power of extract.

**Table 4:** “Adsorption parameters for *CPS* extract on CS surface at different temperatures”.

Temp, °C	Log K_ads_	-ΔG^o^_ads_, kJ mol^─1^	ΔH^o^_ads_, kJ mol^─1^	-ΔS^o^_ads_, J mol^─1^ K^─1^
25	4.92 ± 0.022	38.1 ± 0.1647	27.5	219 ± 0.2333
30	5.69 ± 0.016	43.1 ± 0.1574		233 ± 0.2028
35	5.91 ± 0.017	45.1 ± 0.1321		235 ± 0.1453
40	6.04 ± 0.014	46.7 ± 0.1654		236 ± 0.1732
45	6.35 ± 0.019	49.3 ± 0.1124		241 ± 0.1847

## Electrochemical measurements

### PDP curves

**Fig. 6:**
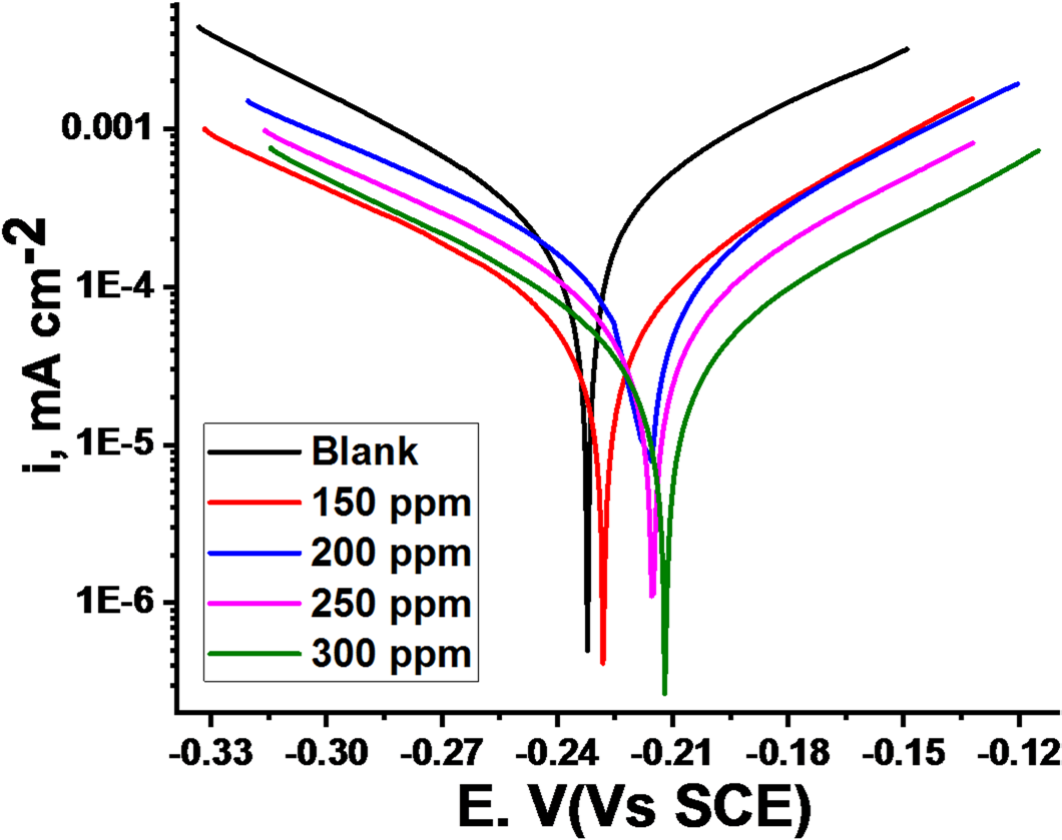
PDP curves at 25 °C for CS in a 1 M HCl solution with and without multiple doses of *CPS* extract.

PDP curves were obtained for carbon steel (CS) in 1 M HCl solution with and without various concentrations of CPS extract at 25 °C (Fig. 6). “A shift of both anodic and cathodic curves to more positive and negative potentials, respectively, indicates the mixed-type inhibitory behavior of the extract. Corrosion current density (i_corr_) and corrosion potential (E_corr_) values were determined from the extrapolation of the linear portions of the polarization curves. These data were subsequently used to investigate the adsorption mechanism. The corrosion rate will be computed using these parameters. The results of electrochemical parameters at different CPS extract doses were recorded in Table 5. The addition of 300 ppm produced the best inhibitory efficiency value of 79.7% (Table 5). “This finding suggests that raising the dose to 300 ppm will effectively slow down the step of CS corrosion”. “The connection between the rise in inhibition efficiency and the fall in current density is linear”. “The adsorption mechanism was examined using the PDP measurement findings”. The anodic (β_a_) and cathodic (β_c_) Tafel slope values are essentially unchanged, as demonstrated in Table 5, resulting in an almost parallel collection of cathodic and anodic lines. Consequently, in these solutions, the adsorbed extract molecules decrease the surface area susceptible to corrosion without affecting the underlying corrosion mechanism of CS^52,53^. Conversely, the E_corr_ data does not show any regular displacement pattern, indicating that the CPS extract functions as a mixed-type inhibitor. According to multiple studies^54^, the behavior of CPS extract as anodic, cathodic, or mixed-type inhibitor depends on the magnitude of E_corr_ displacement relative to the blank”. The extract has an anodic or cathodic function if this displacement beats 85 mV. However, when the displacement is less than 85 mV, as in our case (27 mV), the extract exhibits mixed-type inhibition characteristics. As shown in Table 5, i_corr_ dropped from 205 to 47.4 mA/cm^2^ and the CR altered from 93.8 to 21.8 mpy in the existence of 300 ppm of the extract. It’s owed to the molecules of the extract covering a great portion of the metal surface, impeding its corrosion”. A comparison of PDP curves with and without varying doses of CPS extract reveals that increasing the extract’s dose shifts the E_corr_ towards less negative values and diminishes the anodic process^55^.

**Table 5:** PDP parameters for the dissolution of CS at 25 °C with and without multiple doses of CPS extract.

[Inh] ppm	-E_corr_ mV vs. SCE	i_corr_ µA m^− 2^	β_a_ mV dec^− 1^	- β_c_ mV dec^− 1^	CRx10^3^ mpy	θ	% IE
**Blank**	232 ± 0.1741	58.7 ± 0.1345	88.4 ± 0.1741	118 ± 0.1474	34.7	---	---
150	228 ± 0.1857	13.7 ± 0.1241	85.5 ± 0.1574	116 ± 0.1574	20	0.767	76.7
200	216 ± 0.1987	10.5 ± 0.1245	81.4 ± 0.1144	102 ± 0.1789	15	0.821	82.1
250	215 ± 0.1547	9.3 ± 0.1789	88.3 ± 0.1758	105 ± 0.1578	13	0.842	84.2
300	211 ± 0.1245	5.2 ± 0.1475	87.8 ± 0.1879	91 ± 0.1784	9	0.911	91.1

### EIS measurements

**Fig. 7:**
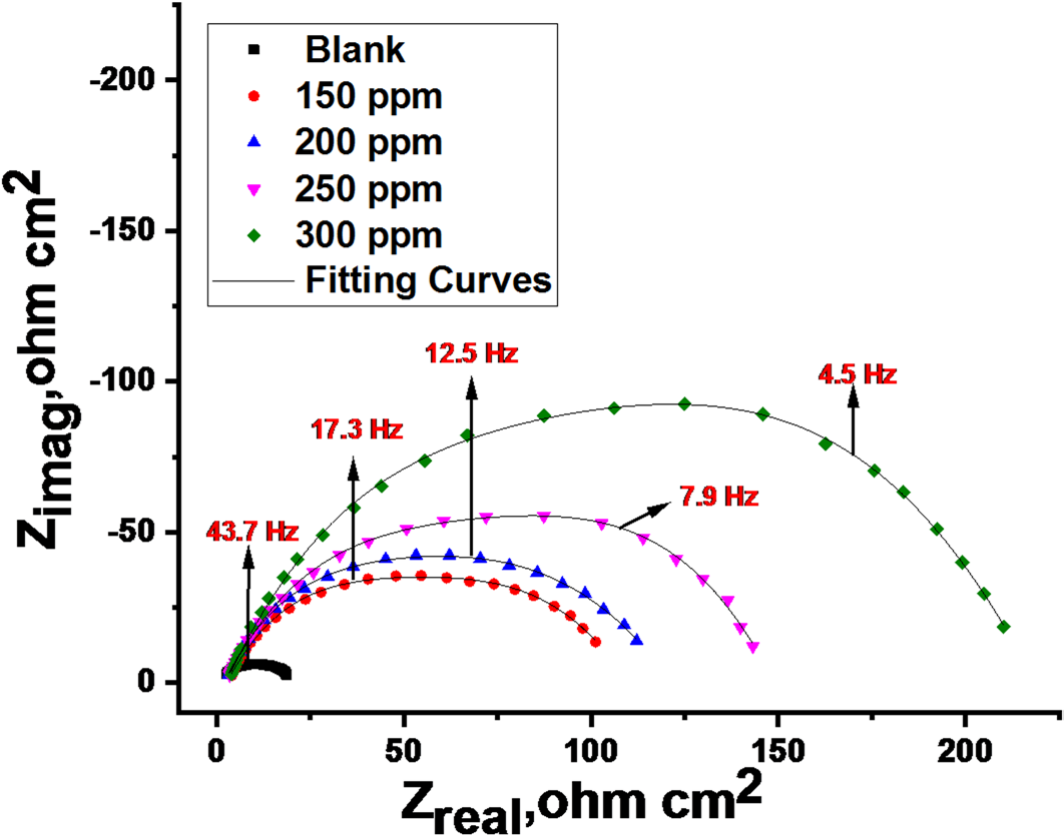
**“**Nyquist diagrams at 25 °C for CS in a 1.0 M HCl solution with and without multiple doses of CPS extract”.

**Fig. 8:**
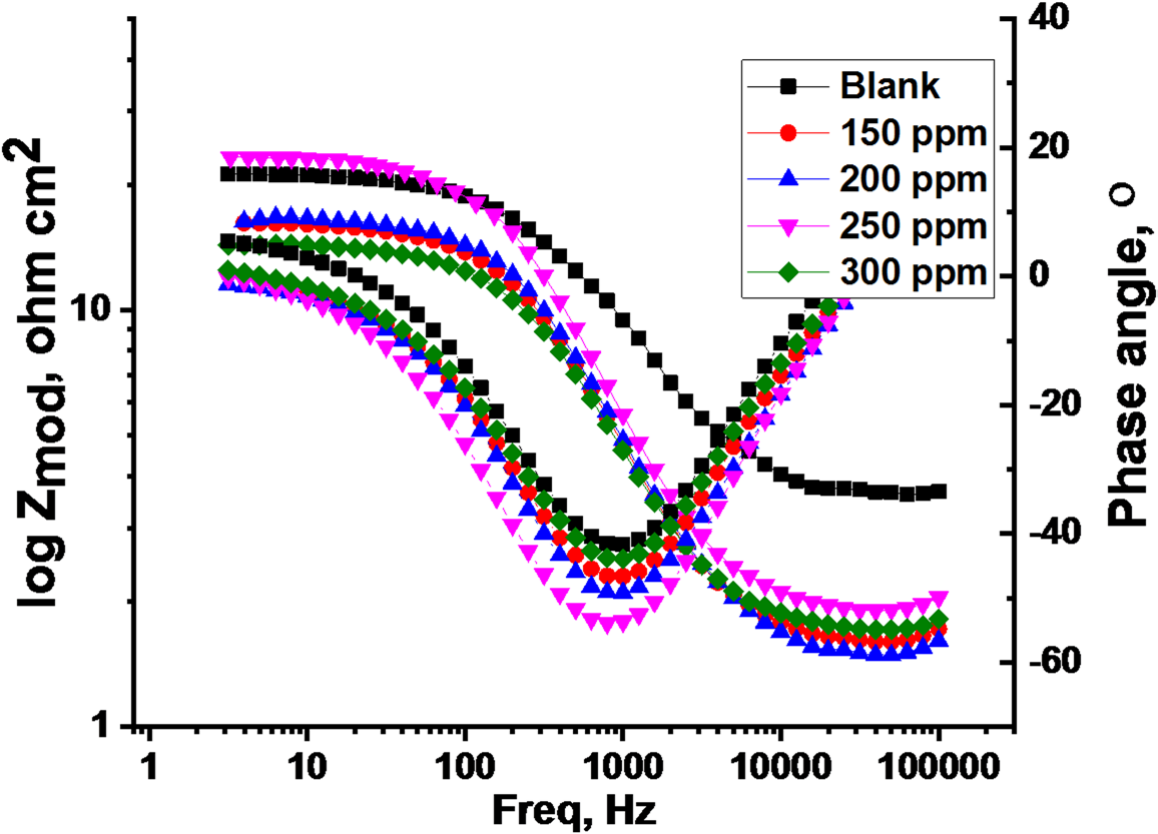
**“**Bode diagrams 25 °C for CS in a 1.0 M HCl solution with and without multiple doses of CPS extract”.

**Fig. 9:**
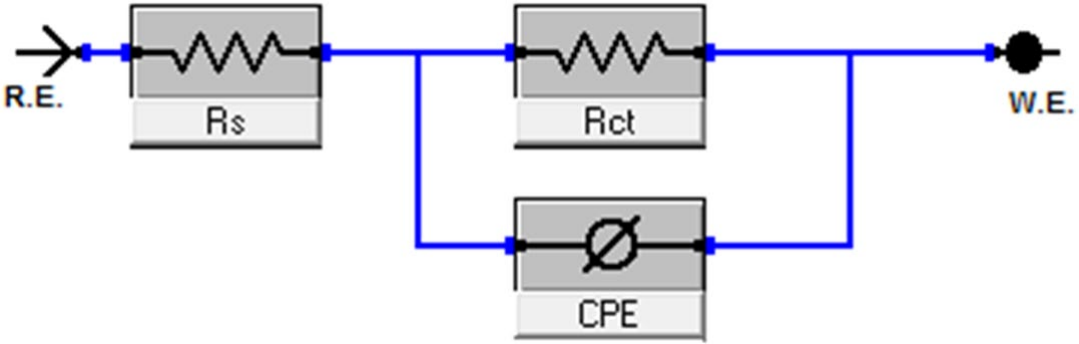
The analogous circuit models.

EIS was employed to investigate the kinetics and surface properties of the carbon steel (CS) electrode. Nyquist and Bode plots obtained at 25 °C (Figs. 7 and 8) illustrate the behavior of CS in 1.0 M HCl solution in the absence and presence of various CPS extract concentrations. “EIS parameters, including double-layer capacitance (C_dl_) and charge transfer resistance (R_ct_), were extracted from the data and are summarized in Table 6. The increasing diameter of the semicircle in the Nyquist plots (Fig. 7) with increasing extract concentration indicates that the corrosion process is predominantly controlled by charge transfer. The semicircular Nyquist plot form (Fig. 7) indicates the roughness and inhomogeneity properties of the electrode surface. “Moreover, the semicircle at the top of the frequency area is related to the time constant, which is also connected to CPE (Y_0_ is its magnitude) which is considered a surface irregularity of the electrode and _ct_. The values of Y_0_ decrease as _dl_ by improving doses of extract. The analogous circuit model (Fig. 9) shows a general instance of the frequency response of an interface defined by charge transfer and diffusion processes. The development of a protective layer on the surface of CS is responsible for the increase in log (Z) values for varying inhibitor concentrations in the Bode diagram (Fig. 8), which also shows a drop in frequency values. However, when compared to the blank (40^o^), the phase angle values rise to a more negative value of roughly 60^o^ for an ideal concentration of 300 ppm, suggesting that the tested inhibitor is effective. Table 6 illustrates that when extract dose rises, R_ct_ values rise and C_dl_ values drop. This is explained by water molecules exchanging with extract-adsorbed molecules and/or thicken the double layer”. Higher R_ct_ values with increasing CPS extract doses are consistent with superior resistance to electrochemical corrosion at elevated extract doses. But when the extract is added to 1.0 M HCl, the “n” values decrease (from 0.985 to 0.977) in comparison to the results obtained in the reference electrolyte (0.991). This means that the “n” value lessens with the increase in extract dose, suggesting that the extract does not adsorb onto the CS surface uniformly, hence the surface is comparatively homogeneous^56,57^. The precision of the fitting results was assessed utilizing the chi-squared method”. “The achieved results for all outcomes reveal that the fitted results strongly concur with the experimental data, as indicated by the modest chi-squared values” (Table 6). “This method’s 83.7% inhibitory efficiency (Table 6) is in good accord with the PDP method”.

**Table 6:** EIS parameters 25 °C for CS in a 1.0 M HCl solution with and without multiple doses of CPS extract.

[Inh] ppm	*n*	Y_o_, (µΩ^−1^ s^*n*^ cm^− 2^)	*R*_ct_ Ω cm^2^	C_dl_ µFcm^− 2^	θ	%IE	Goodness of fit (χ2)
Blank	0.996	417	18 ± 0.2333	408 ± 0.2028	--	--	18.11 × 10^− 3^
150	0.949	399	101 ± 0.1453	335 ± 0.1547	0.822	82.2	16.50 × 10^− 3^
200	0.922	367	112 ± 0.1763	281 ± 0.1624	0.839	83.9	14.28 × 10^− 3^
250	0.915	354	143 ± 0.1453	268 ± 0.1321	0.874	87.4	15.24 × 10^− 3^
300	0.907	293	210 ± 0.1741	221 ± 0.1742	0.914	91.4	19.34 × 10^− 3^

## Examining the surface

### AFM tests

**Fig. 10:**
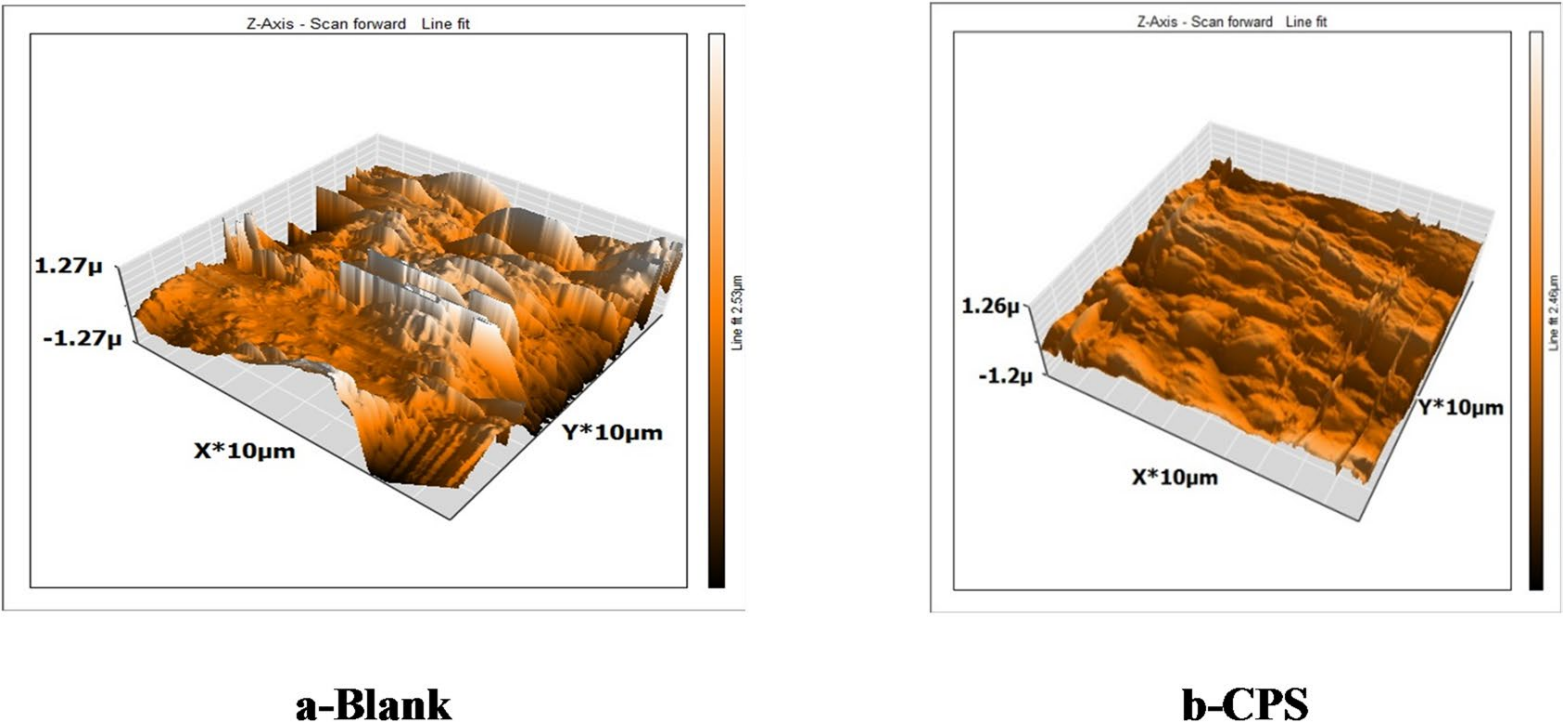
AFM Micrograph for CS in 1 M HCl without (a) and with 300 ppm of *CPS* extract (b).

When discussing the inhibitory effect on the metal/solution interface, “AFM has proven to be a useful instrument for estimating surface morphology investigations^58^. An essential test for determining a metal’s surface roughness at the maximum resolution in nanometer fraction is AFM. This method can provide information about the metal surface’s shape, which is helpful for studies on corrosion science. Figure 10 displays three-dimensional AFM pictures. In contrast, the CS sample treated with 300 ppm CPS has low roughness (147 nm) and a smoother surface when compared to the blank sample”. This is significant because the CPS adsorbed on the surface of the CS metal creating a protective film^59^. The average roughness (R_a_) shows that the metal surface after being submerged in 1.0 M HCl is destroyed and the roughness rises (581 nm) as in Table 7.

**Table 7:** 3D AFM scans of the surface of CS samples with and without CPS extract.

	*R*_man_, µm	RMS roughness (Rq) nm	Mean Roughness Ra), nm
Blank	10.136	764	581
PSC	1.744	179	147

### FT-IR analysis

**Fig. 11:**
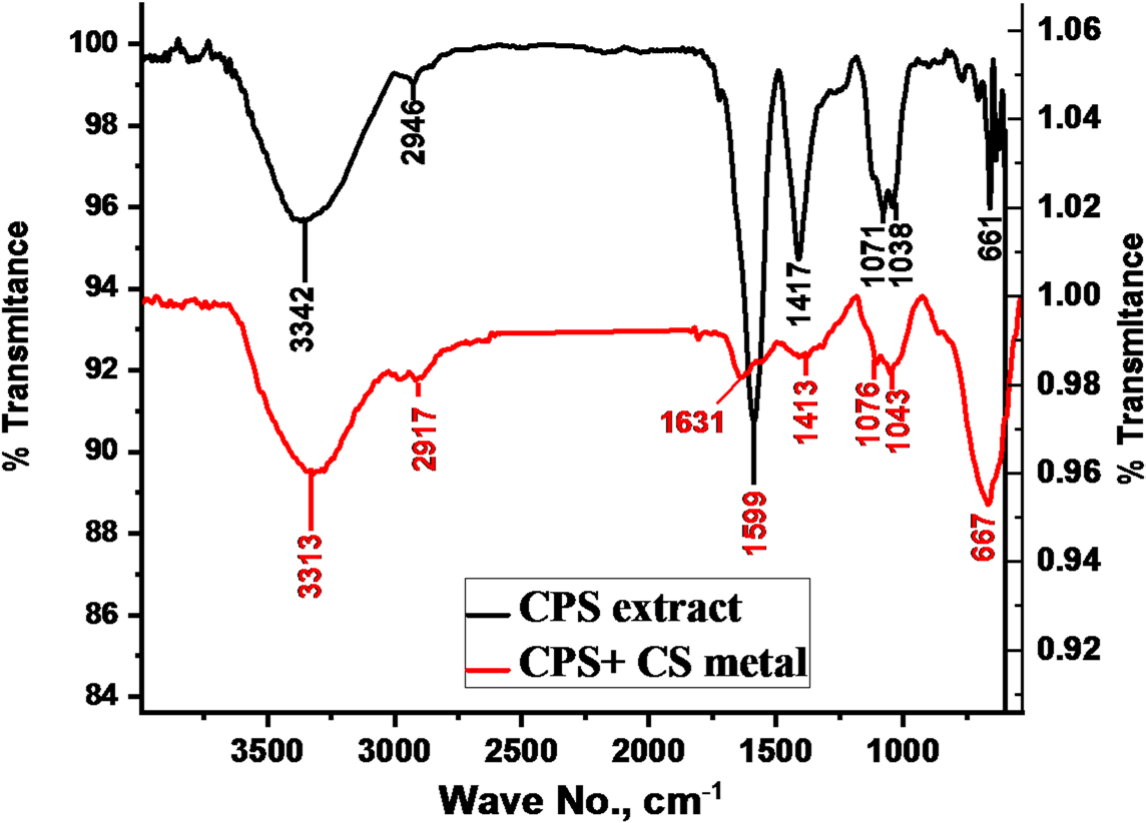
**“**FT-IR spectra of *CPS* extract (black) and the extract adsorbed on the CS surface” (blue).

FT-IR spectroscopy was employed to identify functional groups involved in the formation of the protective layer on the metal surface. Comparative spectra of pure CPS extract and extract adsorbed on the CS surface (Fig. 11) revealed key functional groups. A broad band at 3272 cm⁻¹ indicated the presence of O-H, C-H, and N-H groups, while bands at 2905 cm⁻¹ and 2798 cm⁻¹ corresponded to C-H stretching vibrations. The peak at 1262 cm⁻¹ was attributed to C = O and C-O (acidic) groups, and the band at 1067 cm⁻¹ was assigned to C-O stretching. A shift in peak positions between the spectra of pure and adsorbed extract suggests the involvement of these functional groups in the interaction between CPS and the CS surface^60^.

## Theoretical calculations

### Quantum chemical parameters

**Fig. 12:**
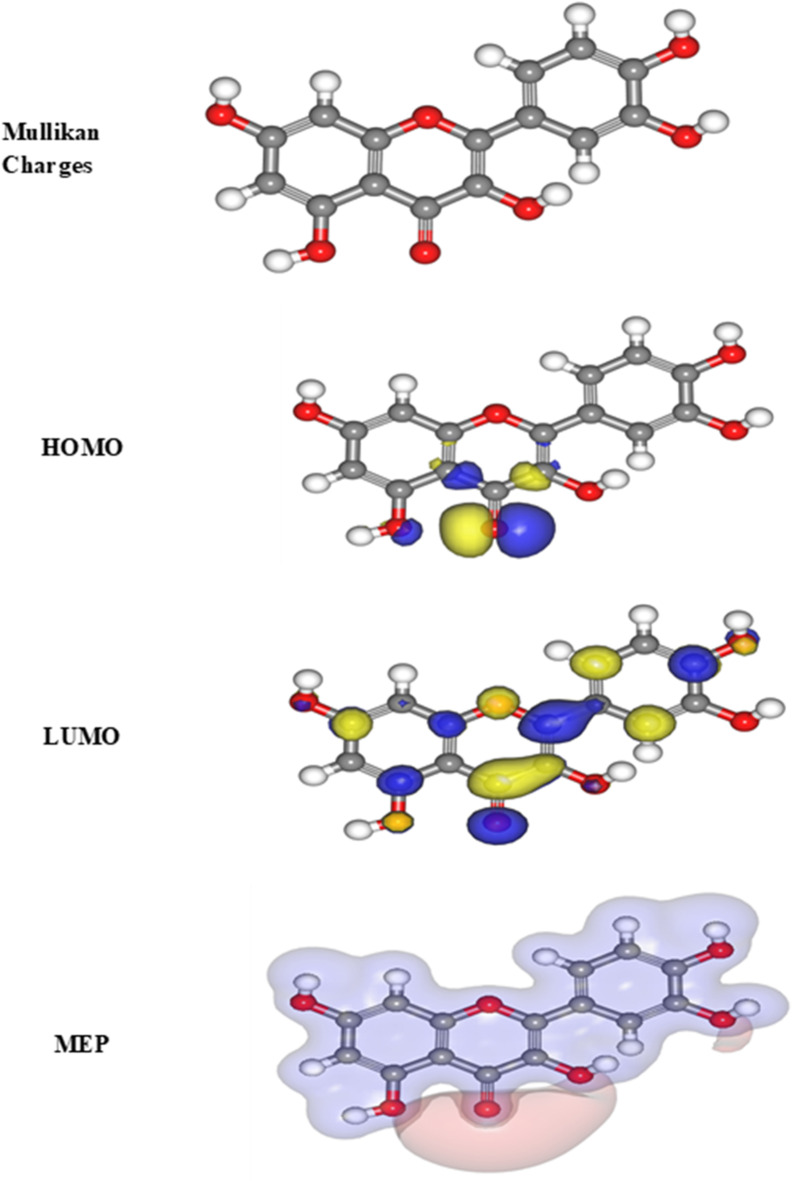
*“*Molecular structure of the investigated drug, and its Frontier molecular orbital density distribution (HOMO and LUMO) and the optimized molecular structure”.

It was found that the content of quercetin varied from 1.7 mg/g to 12.8 mg/g among different parts of caper. So, we try to calculate the DFT and MC for this main component in caper extract (see Fig. S2 and Fig S3) in the supplementary files. According to the DMol3 module built within the Materials Studio version 7.0 software, quantum chemistry was used for all calculations in the current study. “Fig. 12 displays the examination of the inhibitors’ optimal geometry, Molecular electrostatic potential (MEP) map, highest occupied molecular orbital E_HOMO_ denotes the ability of the molecule to donate electron, whereas E_LUMO_ describes the ability of the molecule to accept electron. Thus, the highest value of E_HOMO_ refer to a major affinity for the donation of electrons to unoccupied molecular orbital that was d-orbital of iron atoms and lowest unoccupied molecular orbital (LUMO) density distributions. HOMO and LUMO can determine the donation-acceptance capacity and the molecular reactivity of the inhibitor. The inhibition efficiency increases with an increase in E_HOMO_ values along with a decrease in E_LUMO_ values. The increasing values of E_HOMO_ imply a superior tendency to donate electrons to the molecule with empty orbitals. The dipolar moment (µ) is a measure of the polarity with the covalent bond. The energy band gap ΔE_g_ was defined as: ΔE = E_HOMO_ − E_LUMO_. A smaller ΔE value indicates greater molecular reactivity. Generally, a smaller energy gap means a molecule can more easily donate or accept electrons, which facilitates stronger adsorption onto the metal. Therefore, a lower energy gap value corresponds to a highly reactive molecule with good corrosion inhibition efficiency on the metal surface^61^. Table 8 displays the calculated dipole moments (µ), which indicate the polarity of the covalent bonds within the studied compounds. The higher µ value of inhibitor (15.94 Debye) suggests greater asymmetry in its charge distribution. This larger dipole moment increases inhibitor polarity, leading to stronger electrostatic interactions between the inhibitor and the charged CS surface in acidic HCl media.

**Table 8:** “The quantum parameters for the investigated drug utilized (PM3)”.

Parameters (Variable)	DFT
E_HOMO,_ (ev)	−5.150
E_LUMO,_ (ev)	−3.536
∆E, (eV), (E_L_-E_H_)	1.614
µ (debye), (Dipole moment)	15.94

### Monte Carlo (MC) simulation

**Fig. 13:**
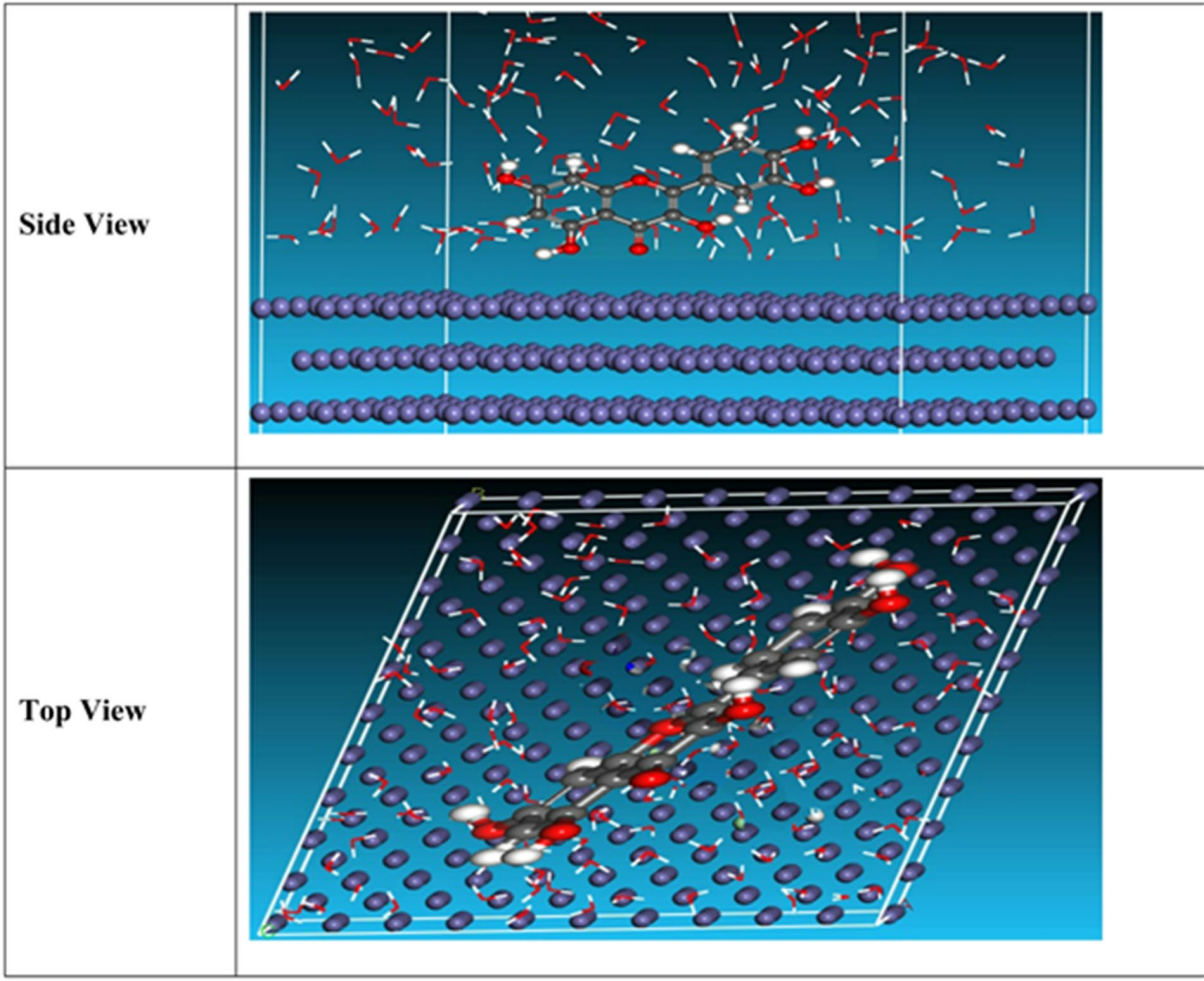
“The inhibitor’s equilibrium adsorption configuration on the surface of Fe (1 1 0): top and side views “.

The side and top observations of the most suitable adsorption formations for the “inhibitor tested on CS surface obtained from the adsorption locator module are thus shown in Fig. 13. Adsorption energy is characterized as declining energy, when materials are mixed during the adsorption process in which an electron, ion, or molecule (adsorbent) is bound to the solid surface. Table 9shows that inhibitor has a greater adsorption energy, indicating that unused inhibitor will heavily adsorb on the toughened surface of CS to form adsorbed stable layers that will prevent corrosion^62^.”.

**Table 9:** Results and descriptors measured by the Monte Carlo simulation for adsorption of inhibitor molecule on Fe (1 1 0).

Structures	Adsorption energy	Rigid adsorption energy	Deformation energy	Compound dE_ad_/dNi	H_2_O dE_ad_/dNi
Fe (1 1 0)/inhibitor/H_2_O	−1566.677	−1621.547	54.87	−78.53	−12.31

This configuration, leading to greater surface coverage than other orientations, improves the overall inhibition efficiency (η). These findings support previous research in this study, which indicates that improved surface coverage results in more effective corrosion protection. Table 9 quantitatively illustrates this interaction. The most favorable adsorption interactions are associated with the lowest adsorption energies, which correspond to the most stable configurations observed in the system. The adsorption energy of inhibitor is calculated as 1566.677 kJ mol^− 1^, led to interact strongly with the surface^63^. The greater magnitude of adsorption energies in the aqueous phase indicates stronger adsorption interactions compared to the gas phase, as solvation effects in water enhance the stabilization of the inhibitor-metal complex. The results demonstrated that inhibitor with its higher dipole moment, lower HOMO-LUMO gap, and greater electron-donating ability, exhibits superior adsorption and interaction with the Fe (1 1 0) surface. This is further supported by the adsorption energies obtained from Monte Carlo simulations,

### Mechanism of corrosion Inhibition

**Fig. 14:**
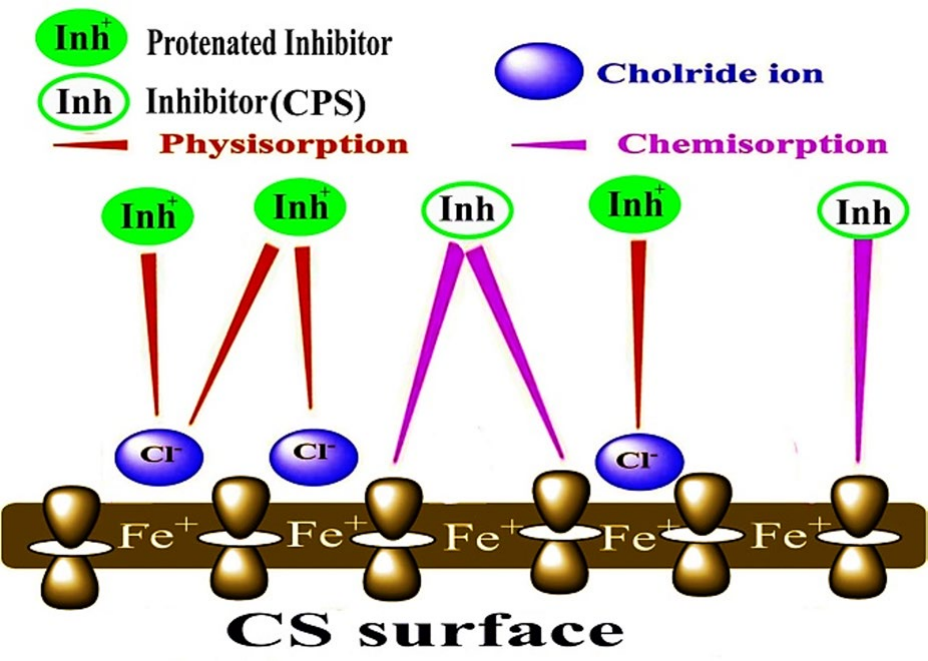
Mechanism of inhibition.

Based on electrochemical and WL studies, “it was found that the examined extract works by adsorbing on the metal surface in accordance with the Temkin isotherm, providing strong protection against corrosion of CS in a 1.0 M HCl solution. The co-adsorption of cationic species and Cl^−^ions may account for the excellent inhibitive qualities of the CPS extract in HCl. Chemisorption and physisorption are the two types of coupled adsorption that are feasible. Both electrically charged metal surfaces and charged species in solution are necessary for physisorption to occur. The electric field’s metal surface charge as it appears at the metal/solution interface. On the other hand, in a chemisorption process, inhibitor molecules may transfer or charge-charge to the metal surface in order to form a coordination bond. It is possible to accomplish this when both positive and negative charges are present on the metal surface. Two different types of inhibitory mechanisms have frequently been proposed. **Kind 1**: the point where Cl^−^ ions have previously adsorbed on the metal surface and extract cations are adsorbed there. In the presence of Cl^−^ ions, extract cations may be able to adsorb due to the creation of an intermediate bridge and the negative ends of the halide metal dipoles facing the solution, which increase the adsorption of the extract cations onto the dipoles^64,65^. This would have the beneficial synergistic effect. This is an example of physisorption for the extract cations on the surface of the chloride bridge produced on the CS. **kind 2**: A large extract cations molecules is present at large extract dose. As a result, Cl^−^ ions may face competition from cationic species for adsorption sites on the CS surface”. In this case, inhibitor species are adsorbed as a result of interactions between donors (π-electrons delocalized within the rings) and recipients (Vacant low-energy d-orbitals of the surface atoms of Fe). The electron density of the rings is greatly increased by the oxygen groups’ capacity to donate electrons^66^. Figure 14 represents the mechanism of inhibition.

## Conclusions

The following conclusions can be drawn from the previous discussion:

1. The results of the study showed that the inhibition process rose with increasing both the concentrations of the extract and the temperature of the medium.
2.A maximum inhibition performance of 95.2% was shown by the extract at concentration 300 ppm in 1 M HCl and at 45 °C.
3.“The adsorption of CPS extract on the CS surface was mainly chemical adsorption and obeyed.

by the Temkin adsorption isotherm.”

4. 4. According to the results of the PDP tests, the CPS extract is categorized as a mixed type inhibitor.
5. 5. The results of EIS showed that the decrease of the C_dl_ values and the increase of the R_ct_ when the concentrations of the CPS was added. That is due to the adsorption of CPS molecules on the metal surface.
6. 6. FTIR spectra provide evidence of the interaction of the interaction between Fe^2+^ and the CPS tested.
7. 7. AFM and SEM-EDX analysis surface studies showed that a protective layer of CPS extract covers the CS surface.
8. 8. What was interesting was that the results in all the methods used were quite consistent with each other.

## Supplementary Information

Below is the link to the electronic supplementary material.

Supplementary Material 1

## Data Availability

The data that support the findings of this study are available from the corresponding author upon reasonable request.
