# Supplementary material for: Insight into the anti-corrosion performance of capparis spinoza extract as a green corrosion inhibitor for carbon steel in hydrochloric acid environment
Source: Sci Rep. 2025 Dec 19;15:44137. doi: 10.1038/s41598-025-30968-5 (PMC12717061; doi:10.1038/s41598-025-30968-5)
Supplement: Supplementary file 1 — Supplementary Material 1 [file 41598_2025_30968_MOESM1_ESM.docx]

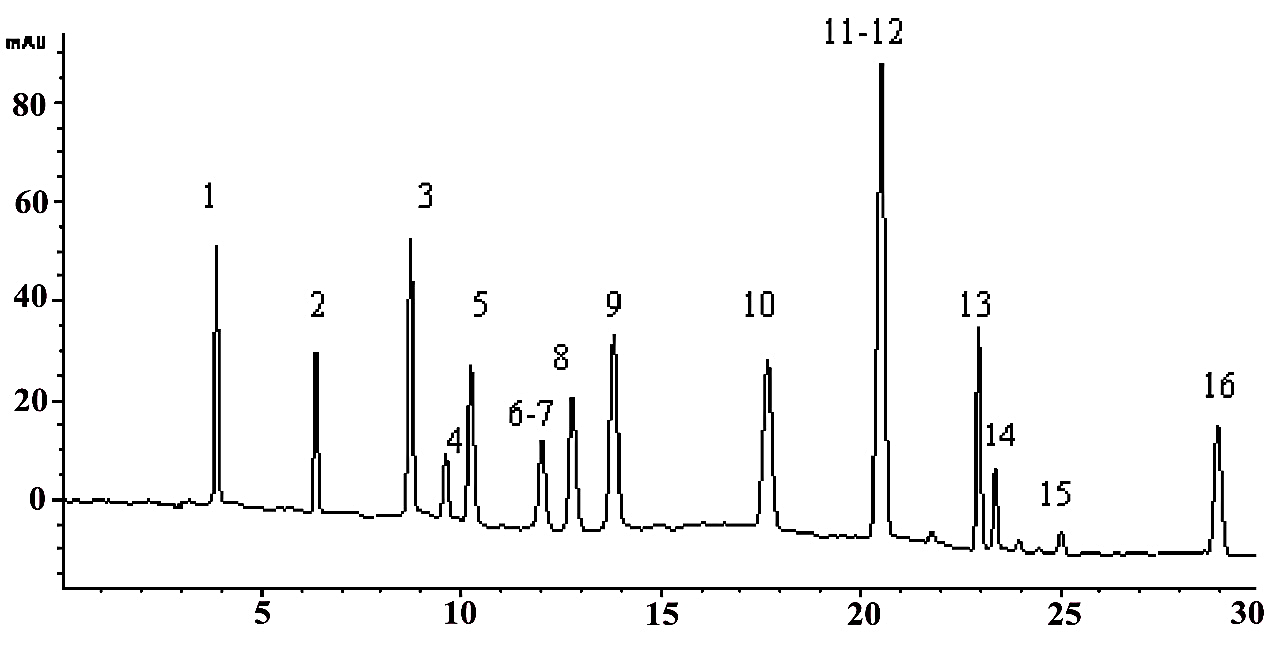


^*^Fig. S1. RP-HPLC chromatogram of phenolic standards at 280 nm. Peak identification: 1, Gallic acid; 2, proto-catechuic acid; 3, protocatechuic aldehyde; 4, gentisic acid; 5, chlorogenic acid; 6, p-OH benzoic acid; 7, vanillic acid; 8, caffeic acid; 9, syringic acid; 10, vanillin; 11, syring aldehyde; 12, p-coumaric acid; 13, ferulic acid; 14, sinapic acid; 15, benzoic acid; 16, rosmarinic acid.


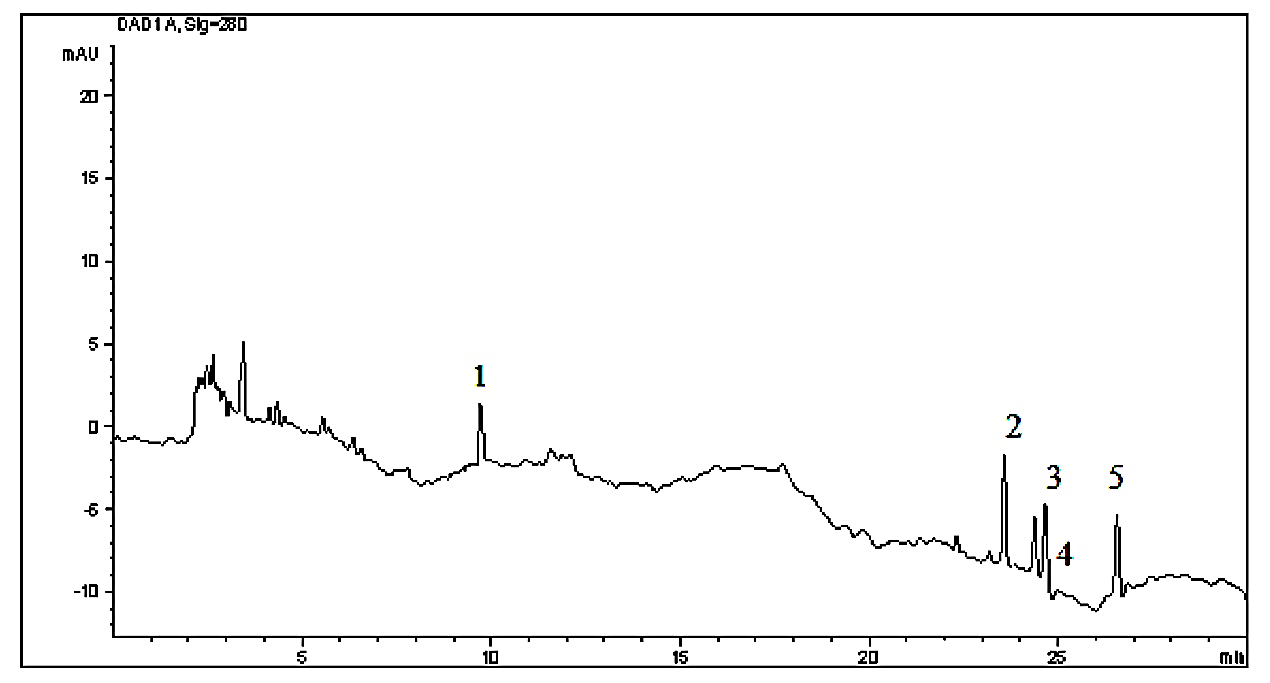


^*^Fig. S2. RP-HPLC chromatogram of Capparis spinosa L. (1 mg/mL) at 280 nm. Peak identification: 1, Gentisic acid; 2, sinapic acid; 3, unknown; 4, benzoic acid; 5, unknown.


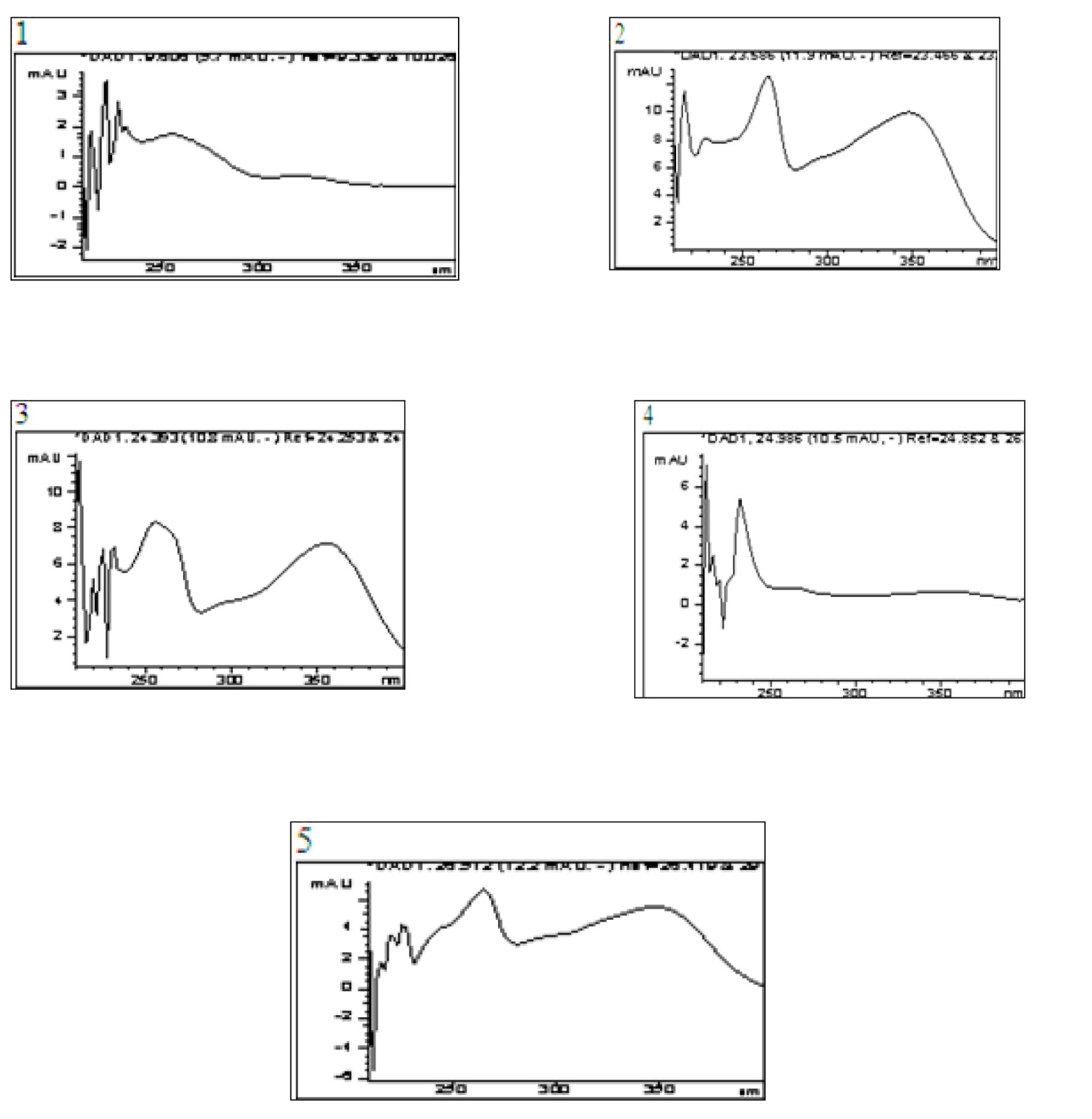


^*^Fig. S3. RP-HPLC-UV spectrums of phenolic component peaks of Capparis spinosa L. (1 mg/mL). Peak identification: 1, Gentisic acid; 2, sinapicacid; 3, unknown; 4, benzoic acid; 5, unknown (200–400 nm)

**Ref. Fig.S1, Fig. S2 and Fig. S3**

**^*^Rezzan Aliyazicioglu, Ozan Emre Eyupoglu, Huseyin Sahin, Oktay Yildiz, Nimet Baltas, Phenolic components, antioxidant activity, and mineral, analysis of *Capparis spinosa* L, Afr. J. Biotechnol. November 2013, 6643-6649, DOI: 10.5897/AJB2013.13241**


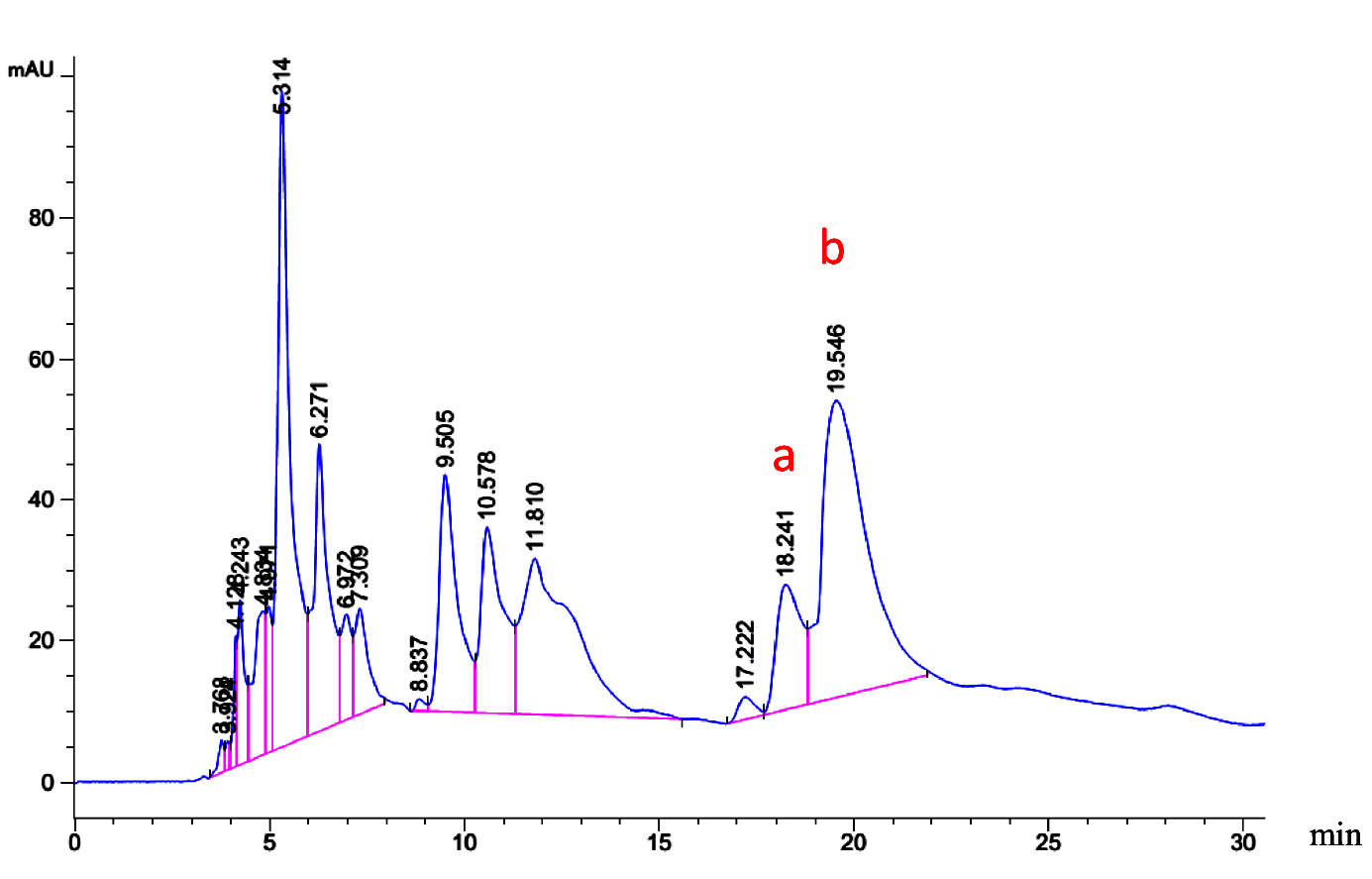


Fig. S4. High-performance liquid chromatography (HPLC) analysis of Capparis spinosa extract (CS) components. HPLC spectrogram of CS (detection at 210 nm).

Fig. S4

**^**^Yina L, Wenhuan Zhang, Lidan Zhou, Yue Xiong, Qing Liu, Xuemei Shi, Jun Tian, The moisturizing effect of *Capparis spinosa* fruit extract, targeting filaggrin synthesis and degradation, *J Cosmet Dermatol.* 2023; 22:651–660, DOI: 10.1111/jocd.15461**
